# Preyssler-type phosphotungstate is a new family of negative-staining reagents for the TEM observation of viruses

**DOI:** 10.1038/s41598-022-11405-3

**Published:** 2022-05-12

**Authors:** Koichi Sahiro, Yasuhiko Kawato, Kanae Koike, Tsuneji Sano, Toshihiro Nakai, Masahiro Sadakane

**Affiliations:** 1 Department of Applied Chemistry, Graduate School of Advanced Science and Engineering, Hiroshima University, 1-4-1 Kagamiyama, Higashi-Hiroshima, 739-8527 Japan; 2 Pathology Division, Nansei Field Station, Fisheries Technology Institute, Japan Fisheries Research and Education Agency, 516-0193 Minami-Ise, Japan; 3 Natural Science Center for Basic Research and Development, Hiroshima University, 1-4-2 Kagamiyama, Higashi-Hiroshima, 739-8527 Japan; 4 Takehara Marine Science Station, Graduate School of Integrated Science for Life, Hiroshima University, Takehara, 725-0024 Japan

**Keywords:** Inorganic chemistry, Materials chemistry, Other nanotechnology, Transmission electron microscopy, Nanoscience and technology

## Abstract

Transmission electron microscopy (TEM) is an essential method in virology because it allows for direct visualization of virus morphology at a nanometer scale. Negative staining to coat virions with heavy metal ions must be performed before TEM observations to achieve sufficient contrast. Herein, we report that potassium salts of Preyssler-type phosphotungstates (K_(15-n)_[P_5_W_30_O_110_M^n+^], M = Na^+^, Ca^2+^, Ce^3+^, Eu^3+^, Bi^3+^, or Y^3+^) are high-performance negative staining reagents. Additionally, we compare the staining abilities of these salts to those of uranyl acetate and Keggin-type phosphotungstate. The potassium salt of Preyssler-type phosphotungstates has the advantage of not requiring prior neutralization because it is a neutral compound. Moreover, the potassium counter-cation can be protonated by a reaction with H^+^-resin, allowing easy exchange of protons with other cations by acid–base reaction. Therefore, the counter-cations can be changed. Encapsulated cations can also be exchanged, and clear TEM images were obtained using Preyssler-type compounds with different encapsulated cations. Preyssler-type phosphotungstates may be superior negative staining reagents for observing virus. Polyoxotungstates (tungsten-oxide molecules with diverse molecular structures and properties) are thus promising tools to develop negative staining reagents for TEM observations.

## Introduction

Observing viral morphology is essential in virology, for which transmission electron microscopy (TEM) is the most widely used technique because it allows direct visualization at the nanometer scale. Currently, advanced TEM techniques such as cryogenic TEM and electron tomography are being rapidly developed for constructing precise three-dimensional images of viruses and small proteins^1–5^, which require expensive TEM equipment and advanced expertise. Thus, methods for simple, rapid, and clear observations using traditional TEM are needed worldwide.

**Figure 1:**
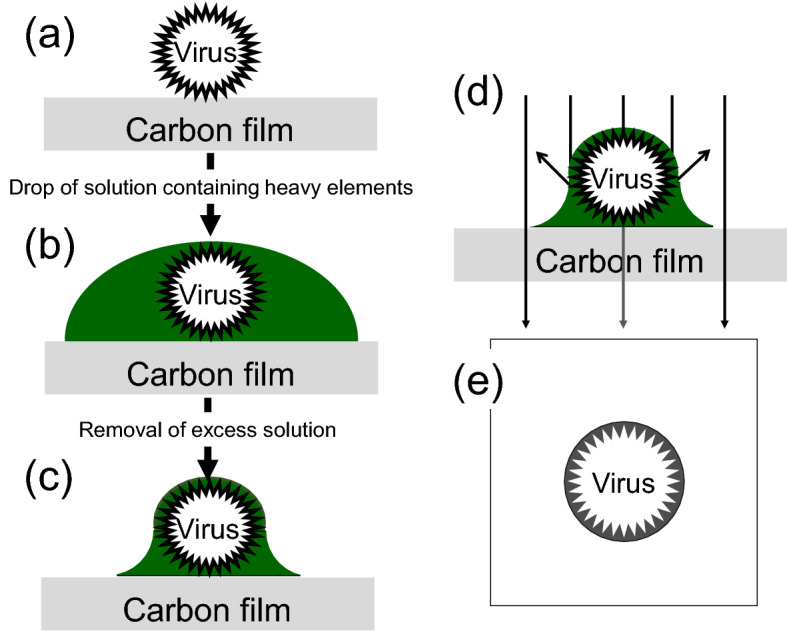
Negative staining method. (**a**) Virions are attached to the carbon support film. (**b**) A solution containing heavy metals (negative-staining reagent) is dropped onto the film. (**c**) Excess solution is removed, and the sample is dried. (**d**) Transmission electron microscopy (TEM) of heavy-metal-coated virions produces (**e**) a reverse-contrast image of the virus.

Generally, negative staining methods using heavy metals are required for traditional TEM observations^6–9^; the procedure is illustrated in Fig. 1. Initially, virions are adsorbed on a carbon support film (Fig. 1a). A small drop of staining reagent containing heavy metals is dropped onto the film (Fig. 1b). After removing excess solution, the sample is dried, leading to coating of the virions by heavy metals (Fig. 1c). Finally, reverse-contrast images are generated via enhanced electron scattering from the heavy elements coating the virions (Fig. 1d,e).

Without using staining reagents, it is difficult to obtain sufficient contrast between the virions and carbon support, resulting in unclear observations of virus morphology because the fragments of virions dispersed on the carbon support film are smaller than the thickness of the film. Therefore, viruses must be coated with heavy metals that have high electron-scattering constants.

**Figure 2:**
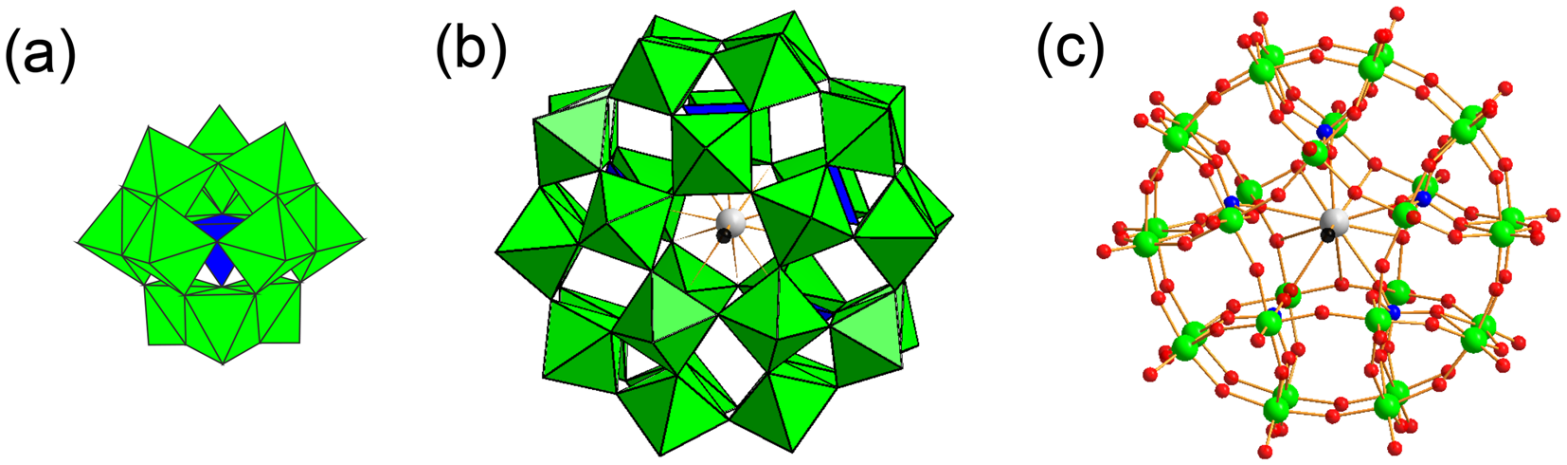
(**a**) Polyhedral representation of Keggin-type phosphotungstate ([PW_12_O_40_]^3−^). (**b**) Polyhedral and (**c**) ball-and-stick representation of Preyssler-type phosphotungstate, ([P_5_W_30_O_110_M^n+^]^(15−n)−^). Blue and green polyhedra represent tetragonal PO_4_ and octahedral WO_6_, respectively. Blue, green, grey, red, and black balls represent P, W, encapsulated cation M (Na^+^, Ca^2+^, Bi^3+^, Y^3+^, or Eu^3+^), O, and O (H_2_O), respectively.

Uranyl acetate ((CH_3_CO_2_)_2_UO_2_) is among the most commonly used negative staining reagents^6–9^. However, because uranyl compounds are internationally controlled nuclear materials, their purchase and storage entail complicated procedures^10,11^. Therefore, alternatives to uranyl acetate must be developed. Phosphotungstic acid (PTA) is a substitute, and the commercially used one is Keggin-type PTA (H_3_PW_12_O_40_) (Supplementary Figs. S1 and S2). It is a protonated Keggin-type phosphotungstate ([PW_12_O_40_]^3^): a ball-shaped molecule having one central tetragonal PO_4_ unit surrounded by 12 octahedral WO_6_ units with *T*_*d*_ symmetry (Fig. 2a)^12^. Keggin-type PTA is a highly acidic compound and is mainly used after neutralization with NaOH or KOH^6–9^. TEM images obtained using this PTA are less clear than those obtained using uranyl acetate. Therefore, other tungsten reagents such as sodium silicotungstate and methylamine tungstate^13^ have been considered as alternative negative-staining reagents^7^.

These tungsten reagents, along with Keggin-type phosphotungstate, belong to the class of polyoxotungstates, which are anionic tungsten oxide clusters^12^. Polyoxotungstates have diverse molecular structures and physico-chemical properties such as stability, solubility, acidity, and crystallinity. Based on this information, we examined a new high-performance negative staining reagent using members of this family of compounds.

Preyssler-type phosphotungstate is a doughnut-shaped molecule with one encapsulated cation (M = Na^+^, Ca^2+^, Eu^3+^, Bi^3+^, or Y^3+^) and 5 tetragonal PO_4_ surrounded by 30 octahedral WO_6_ with *C*_*5v*_ symmetry (Fig. 2b,c). Preyssler-type phosphotungstate is stable over a wide pH range (pH 1–12) and is produced as a potassium salt; thus, it can be used without neutralization^14^. We previously reported that Preyssler-type phosphotungstate (K_12_[P_5_W_30_O_110_Eu]) can be used as a negative staining reagent to observe the approximately 9-nm-thick fimbriae of a bacterium (*Edwardsiella tarda*)^15^. In the current study, we demonstrate that Preyssler-type polyoxotungstates (K_(15-n)_[P_5_W_30_O_110_M^n+^], M = Na^+^, Ca^2+^, Eu^3+^, Bi^3+^, or Y^3+^) are high performance negative-staining reagents for visualizing viruses, which are much smaller than bacteria. We observed the stained virion samples using TEM, scanning electron microscopy (SEM), and atomic force microscopy (AFM) to determine the staining ability of each negative staining reagent.

## Results and discussion

### Comparison of negative-staining reagents

**Figure 3:**
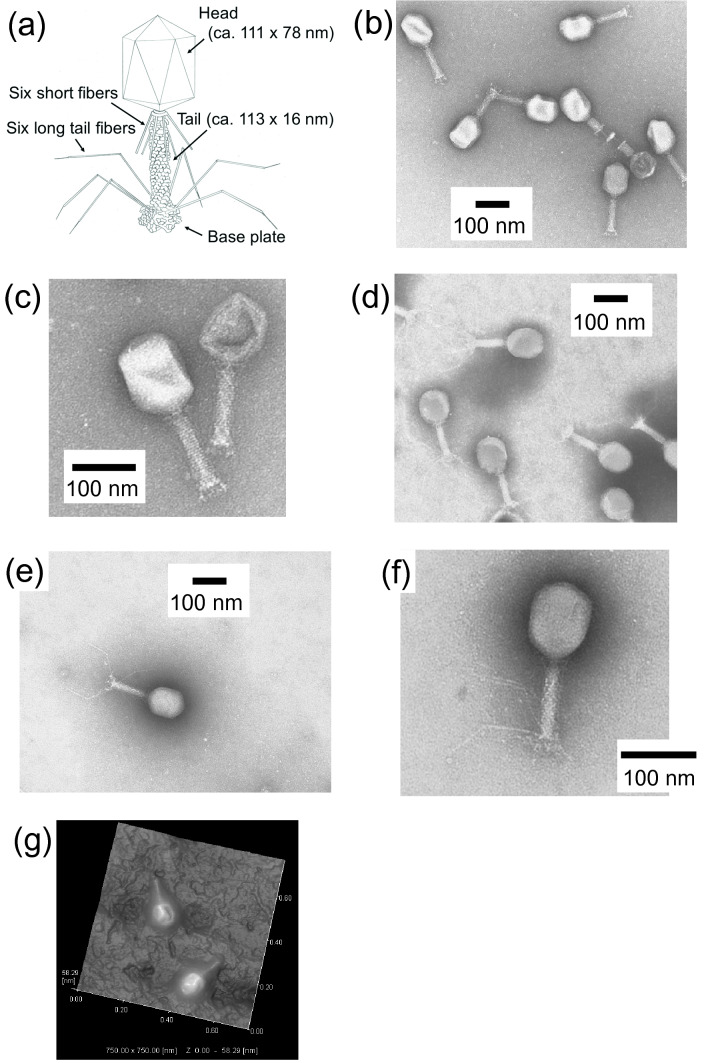
(**a**) Morphology of T4 phage. (**b**–**e**) Transmission electron microscopy (TEM) images of T4 phages using negative staining reagents (**b**, **c**) uranyl acetate, (**d**) neutralized Keggin-type PTA (H_3_[PW_12_O_40_]–KOH), and (**e**, **f**) potassium salt Na-encapsulated Preyssler-type phosphotungstate (K_14_[P_5_W_30_O_110_Na(H_2_O)]). (**g**) Atomic force microscopy (AFM) image of the TEM grid.

Figure 3 shows the TEM images of T4 phages obtained using two common negative staining reagents, uranyl acetate and neutralized Keggin-type PTA, and the potassium salt of Na-encapsulated Preyssler-type phosphotungstate ((K_14_[P_5_W_30_O_110_Na]) as negative staining reagents. Enterobacteria phage T4 (family *Myoviridae*) was selected as a model virus because its detailed morphology has been established^16^. The T4 phage is constructed from a head with an elongated icosahedron shape, tail part, and base plate. In addition, it has six whisker-like short fibers and long tail fibers (Fig. 3a)^17^. Although the head and tail part were clearly visible using uranyl acetate, the short and long fibers were not observed (Fig. 3b,c), which are similar to reported TEM images^18–21^. The head, tail part, and long tail fibers were observable using the neutralized Keggin-type PTA; however, the background was not homogeneous (Fig. 3d). In contrast, the background, head, tail part, and long tail fibers were all clearly observed using the potassium salt of Na-encapsulated Preyssler-type phosphotungstate (Fig. 3e,f). It has been reported that the long tail fibers were also observed by using uranyl acetate^17,22^. However, it is worth to note that we could observe clear images without radioactive uranyl acetate.

### Concentration effect of staining reagents

The concentration of K_14_[P_5_W_30_O_110_Na(H_2_O)] in the negative staining solution is an important factor affecting image clarity. We examined the concentration effect of K_14_[P_5_W_30_O_110_Na(H_2_O)] (Supplementary Fig. S3) and the neutralized Keggin-type PTA (Supplementary Fig. S4) in the staining solution. When the concentration of K_14_[P_5_W_30_O_110_Na(H_2_O)] was high (for example, 2.0 wt%), crystal-like plates of staining reagent were observed, and the phage shape was unclear. Moreover, crystal formation was observed in the SEM image. These images were categorized as image A. Only the heads were observable in image A, which was obtained from approximately 57% of the TEM grid area (Supplementary Fig. S3). From the other 40% of the grid, we obtained images categorized as image B, in which staining reagents coated the T4 phages and parts of the carbon film. In image B, no long tail fibers were observed. However, instances of staining reagents coating T4 phages and clear phage images from only a few percent of the grid were obtained (categorized as image C). In image C, the long tail fibers were clearly observable. The concentration of Preyssler compound in the staining solution was directly proportional to the area where image A was observed and inversely proportional to the areas of images B and C. Moreover, the area ratio of image C reached a maximum when the concentration was 0.3 wt%. However, an area with low contrast was also observed (categorized as image D) that was inversely proportional to the concentration.

Furthermore, an AFM image obtained from image area C on the TEM grid (Fig. 3g and Supplementary Fig. S5) after TEM observation revealed head and tails. The observed length (230 and 225 nm) of the T4 body (head and tail together) was close to that expected for a T4 phage (Fig. 3a). However, the observed height of the head (47 nm) (Supplementary Fig. S5) was less than its thickness (78 nm in Fig. 3a), indicating that the head shrank under TEM vacuum conditions.

In the case of the neutralized Keggin-type PTA (Supplementary Fig. S4), no crystal formation was observed in the high-concentration samples. Although decreasing the concentration improved the images, better images were obtained using Preyssler-type phosphotungstate.

### Difference between Preyssler-type and Keggin-type phosphotungstates

Keggin-type and Preyssler-type phosphotungstates exhibit greatly different stabilities in neutral solution. Supplementary Fig. S6 shows the pH titration curves of Keggin-type PTA and Na-encapsulated Preyssler-type PTA in aqueous solution. For Preyssler-type PTA, the pH rapidly increased when equal moles of NaOH were added to 14 protons, indicating that the Preyssler-type phosphotungstate was stable in aqueous solution with a pH range of 1–12. The reaction in question is as follows:

1 $${\text{H}}_{{{14}}} \left[ {{\text{P}}_{{5}} {\text{W}}_{{{3}0}} {\text{O}}_{{{11}0}} {\text{Na}}} \right] + {\text{14NaOH}} = {\text{Na}}_{{{14}}} \left[ {{\text{P}}_{{5}} {\text{W}}_{{{3}0}} {\text{O}}_{{{11}0}} } \right] + {\text{14H}}_{{2}} {\text{O}}$$

In contrast, the Keggin-type PTA solution remained acidic (pH almost unchanged) after adding equal moles of NaOH to 3 acidic protons. This result indicates that the Keggin-type phosphotungstate molecule ([PW_12_O_40_]^3−^) was decomposed. Moreover, it has been reported that [PW_12_O_40_]^3−^ is stable only under very acidic conditions (pH < 2), and its neutralization produces a complex mixture of phosphotungstate and tungstate species depending on the solution pH^23,24^. In an aqueous solution of pH 7, the main phosphotungstate species detected by phosphorus-31 nuclear magnetic resonance (^31^P NMR) was mono-defective (lacunary) phosphotungstate ([PW_11_O_39_]^7−^) (Supplementary Fig. S2), in which the one moiety [W = O]^4+^ removed from [PW_12_O_40_]^3−^ forms tungsten oxide clusters:

2 $${\text{H}}_{{3}} \left[ {{\text{PW}}_{{{12}}} {\text{O}}_{{{4}0}} } \right] + {\text{7NaOH}} = {\text{Na}}_{{7}} \left[ {{\text{PW}}_{{{11}}} {\text{O}}_{{{39}}} } \right] + {\text{H}}_{{2}} \left[ {{\text{WO}}_{{4}} } \right]\left( {\text{tungsten oxide cluster}} \right) + {\text{4H}}_{{2}} {\text{O}},$$

These species may produce an inhomogeneous background. In contrast, the Preyssler-type phosphotungstate molecule is stable over a wide range (pH 1–12), which might be attributable to the homogeneous background.

### Further advantages of Preyssler-type compounds

Preyssler-type phosphotungstates have several advantages. They are prepared as a potassium salt (K_14_[P_5_W_30_O_110_Na]), which is a neutral compound, and therefore do not require prior neutralization. Moreover, the potassium counter-cation can be protonated by a reaction with H^+^-resin,

3 $${\text{K}}_{{{14}}} \left[ {{\text{P}}_{{5}} {\text{W}}_{{{3}0}} {\text{O}}_{{{11}0}} {\text{Na}}} \right] + {\text{14 H}}^{ + }-{\text{resin}} = {\text{H}}_{{{14}}} \left[ {{\text{P}}_{{5}} {\text{W}}_{{{3}0}} {\text{O}}_{{{11}0}} {\text{Na}}} \right] + {\text{14 K}}^{ + }-{\text{resin}},$$

allowing for easy exchange of protons with other cations by the acid–base reaction

4 $${\text{H}}_{{{14}}} \left[ {{\text{P}}_{{5}} {\text{W}}_{{{3}0}} {\text{O}}_{{{11}0}} {\text{Na}}} \right] + {\text{14AOH}} = {\text{A}}_{{{14}}} \left[ {{\text{P}}_{{5}} {\text{W}}_{{{3}0}} {\text{O}}_{{{11}0}} {\text{Na}}} \right] + {\text{14H}}_{{2}} {\text{O}},$$

where (A = Li^+^, Na^+^, NH_4_^+^, Bu_4_N^+^, and Bu_4_P^+^). Thus, it is possible to change the counter-cations. Clear images with a homogeneous background were obtained using the salts of lithium (Li_14_[P_5_W_30_O_110_Na]), sodium (Na_14_[P_5_W_30_O_110_Na]), and ammonium ((NH_4_)_14_[P_5_W_30_O_110_Na]) (Supplementary Fig. S7). However, long tail fibers were not obtained using tetrabutylammonium ((Bu_4_N)_14_[P_5_W_30_O_110_Na]) and tetrabutylphosphonium ((Bu_4_P)_14_[P_5_W_30_O_110_Na]) salts (Supplementary Fig. S7).

**Figure 4:**
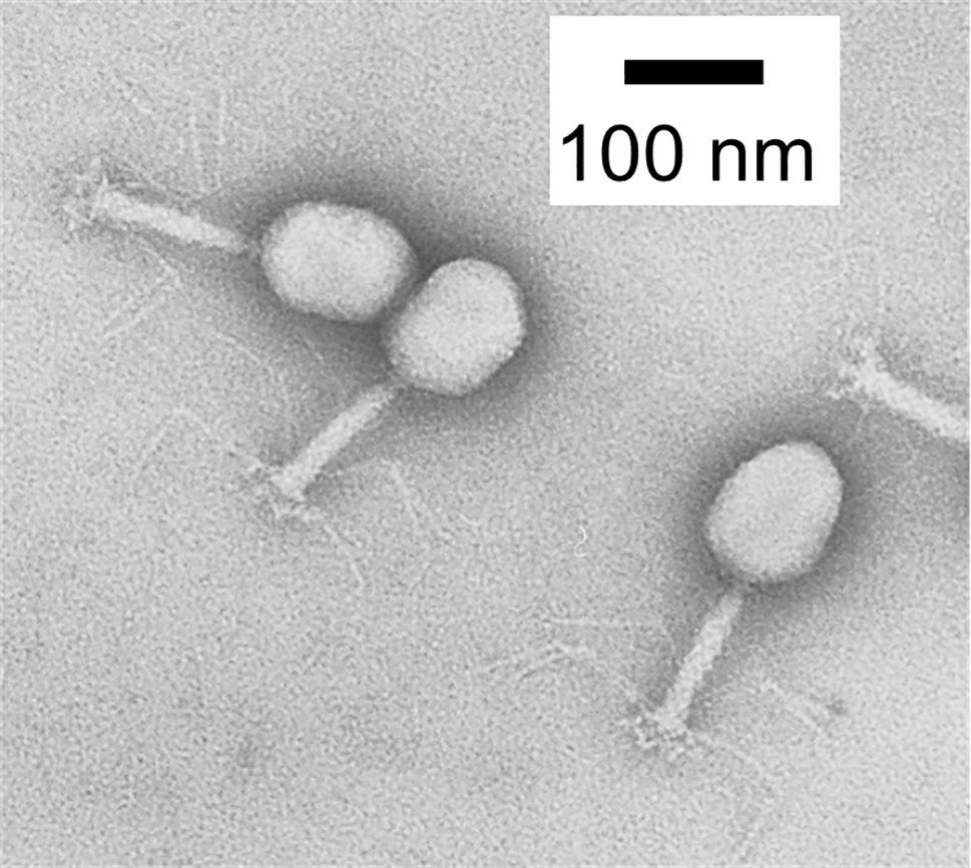
Transmission electron microscopy (TEM) images of T4 phages using Preyssler-type phosphotungstate with encapsulated cation Eu^3+^, K_12_[P_5_W_30_O_110_Eu(H_2_O)], as a negative staining reagent. Concentration of the staining reagent was 0.3 wt% in water.

Furthermore, the encapsulated Na^+^ is exchangeable with other cations that have different charges, such as Ca^2+^, Bi^3+^, Y^3+^, and lanthanoid cations. Such exchange alters the negative charge of the Preyssler molecule without affecting its shape. The change in the negative charge affects the crystallinity of Preyssler molecules and their interaction with the virus surface and carbon film support, changing the performance of the negative staining reagent. Clear TEM images were obtained using these Preyssler-type compounds with different encapsulated cations, such as Ca^2+^^25^, Y^3+^^25^, Bi^3+^^26^, Ce^3+^^27^, and Eu^3+^^25^ (Supplementary Fig. S8). The Eu^3+^-encapsulated compound (K_12_[P_5_W_30_O_110_Eu(H_2_O)]) was the best negative staining reagent among these compounds (Fig. 4) and clear images were obtained from more than 75 area% of grid (Supplementary Fig. S8g).

### Staining performance with other viruses

**Figure 5:**
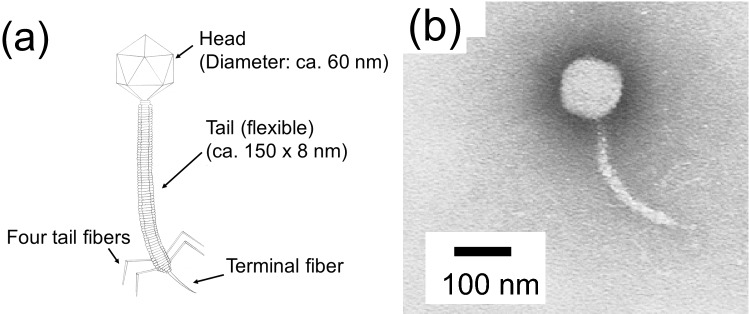
(**a**) Morphology of the lambda phage. (**b**) Transmission electron microscopy (TEM) image of lambda phage using Preyssler-type phosphotungstate with encapsulated cation Eu^3+^, K_12_[P_5_W_30_O_110_Eu(H_2_O)], as a negative staining reagent. Concentration of the staining reagent was 0.3 wt% in water.

**Figure 6:**
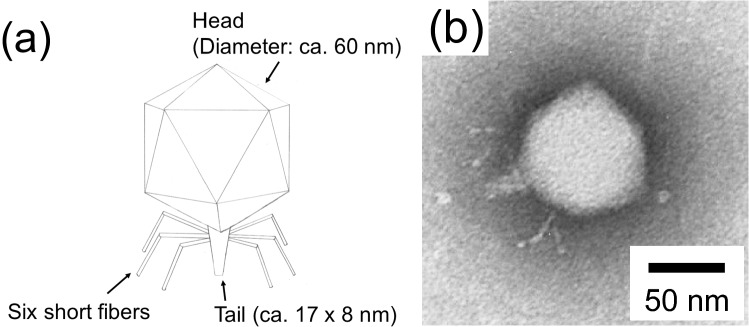
(**a**) Morphology of the T7 phage. (**b**) Transmission electron microscopy (TEM) images of T7 phage using Preyssler-type phosphotungstate with encapsulated cation Eu^3+^, K_12_[P_5_W_30_O_110_Eu(H_2_O)], as a negative staining reagent. Concentration of staining reagent was 0.3 wt% in water.

The Preyssler-type phosphotungstate was a good negative staining reagent for T4 and other phages examined in the present study. The lambda phage (family *Siphoviridae*) has an icosahedral head with a diameter of ca. 60 nm, a long flexible tail with a length of ~ 150 nm, a short terminal fiber, and four tail fibers^17,28^, which were all observed (Fig. 5). The T7 phage (family *Podoviridae*) has an icosahedral head with a diameter of ~ 60 nm, a short tail, and six short fibers^17,29^, which were clearly visible (Fig. 6).

## Conclusions

Negative staining has been widely used to observe the morphologies of viruses^6^, other biological particles, lipid vesicles, micelles, liposomes, and polymer particles^30^. Our results indicate that Preyssler-type phosphotungstates are good negative staining reagents for virus observations. Furthermore, tungsten forms a variety of metal oxide clusters known as polyoxotungstate in an aqueous solution, depending on the other elements present and pH^12^. Polyoxotungstates are promising tools for developing negative staining reagents for TEM observations.

## Methods

### Materials

The potassium salt of Preyssler-type phosphotungstate with different encapsulated cations (K_(15-n)_[P_5_W_30_O_110_M^n+^], M = Na^+^, Ca^2+^, Bi^3+^, Y^3+^, Eu^3+^), and Preyssler-type phosphotungstic acid with encapsulated sodium, (H_14_[P_5_W_30_O_110_Na]), were prepared and purified according to a previously reported method^25^. The obtained potassium salts were dissolved in water and used as the staining solution. An aqueous solution of uranyl acetate (3 wt%) and commercial phosphotungstic acid (TAAB Laboratories Equipment Ltd., Berks, England) were used for comparison. The phosphotungstic acid solution was neutralized using 2 M KOH solution.

### Preparation of phages

Enterobacteria phages T4 (NBRC20004), T7 (NBRC20007), and lambda (NBRC20016) and their host bacteria *Escherichia coli* (NBRC13168 and NBRC12713) were obtained from the NITE Biological Resource Center (NBRC, Chiba, Japan). The phages were propagated by the agar overlay method^31^ using peptone yeast medium (1% polypeptone, 0.2% yeast extract, and 0.1% MgSO_4_·7H_2_O) and purified as previously reported^32^. Additionally, the phage titers (PFU mL^−1^) of the purified phage were determined by the agar overlay method.

### Virus observation

For TEM analysis, the phage solution (10^11^ PFU mL^−1^, 5 μL) was placed in contact with a glow-discharged (JEOL HDT-400, Tokyo, Japan) carbon-coated collodion film on a Cu grid (Nisshin EM, Tokyo, Japan) for 3 min. Excess solution was removed using a filter paper. Subsequently, a drop (5 μL) of staining solution was placed on the grid for 3 min. The staining solution was removed using a filter paper, and the grid was air-dried. TEM (JEOL, JEM-1200EX) with a tungsten filament was employed at 80 kV. The sample grid prepared for the TEM observation was fixed using a carbon tape on a sample holder and observed using SEM (S-4800, Hitachi, Tokyo, Japan) and AFM (SPM-9600, Shimadzu, Kyoto, Japan).

## Supplementary Information

Supplementary Figures.

## Data Availability

All supporting data are found in the supplementary information.

## References

[1] Vanhecke D (2011). Cryo-electron tomography: Methodology, developments and biological applications. J. Microsc..

[2] Henderson R (2018). From electron crystallography to single particle cryoEM (Nobel lecture). Angew. Chem. Int. Ed..

[3] Frank J (2018). Single-particle reconstruction of biological molecules-story in a sample (Nobel lecture). Angew. Chem. Int. Ed..

[4] Dubochet J (2018). On the development of electron cryo-microscopy (Nobel lecture). Angew. Chem. Int. Ed..

[5] Fernandez-Leiro R, Scheres SHW (2016). Unravelling biological macromolecules with cryo-electron microscopy. Nature.

[6] De Carlo S, Harris JR (2011). Negative staining and cryo-negative staining of macromolecules and viruses for TEM. Micron.

[7] Scarff CA, Fuller MJG, Thompson RF, Iadanza MG (2018). Variations on negative stain electron microscopy methods: Tools for tackling challenging systems. J. Vis. Exp..

[8] Ackermann H-W, Clokie MRJ, Kropinski AM (2009). Basic phage electron microscopy. Bacteriophages: Methods and Protocols Vol. 1: Isolation, Characterization, and Interactions.

[9] Ackermann H-W, Heldal M, Wilhelm SW, Weinbauer MG, Suttle CA (2010). Basic electron microscopy of aquatic viruses. Manual of Aquatic Viral Ecology.

[10] United States Nuclear Regulatory Commission. General Licence Uses of Nuclear Materials. https://www.nrc.gov/materials/miau/general-use.html (2020). Accessed 28 Apr 2022.

[11] Nuclear Regulation Authority [Japan]. Act on the Regulation of Nuclear Source Material, Nuclear Fuel Material and Reactors (June 10, 1957, amended Nov. 22, 2013), Chapter VI-2. English translation. https://www.oecd-nea.org/law/legislation/jpn-material-reactors.pdf (2014). Accessed 28 Apr 2022.

[12] Pope MT (1983). Heteropoly and Isopoly Oxometalates.

[13] Sukmana NC, Sugiarto ZZ, Sadakane M (2021). Structure and thermal transformations of methylammonium tungstate. Z. Anorg. Allgem. Chem..

[14] Alizadeh MH, Harmalker SP, Jeannin Y, Martic-Frère J, Pope MT (1985). A heteropolyanion with fivefold molecular symmerty that contains a nonlabile encapsulated sodium ion. The structure and chemistry of [NaP_5_W_30_O_110_]^14^^−^. J. Am. Chem. Soc..

[15] Istiqomah I (2015). Fimbriae expression by *Edwardsiell tarda* in high-salt culture conditions. Fish Pathol..

[16] Yap ML, Rossmann MG (2014). Structure and function of bacteriophage T4. Future Microbiol..

[17] King AMQ, Adams MJ, Carstens EB, Lefkowitz EJ (2011). Virus Taxonomy: Ninth Report of the International Committee on Taxonomy of Viruses.

[18] Nakakoshi M, Nishioka H, Katayama E (2011). New versatile staining reagents for biological transmission electron microscopy that substitute for uranyl acetate. J. Electron Microsc..

[19] Hosogi N, Nishioka H, Nakakoshi M (2015). Evaluation of lanthanide salts as alternative stains to uranyl acetate. Microscopy.

[20] Zhao J (2019). Characterizing the biology of lytic bacteriophage vB_EaeM_phiEap-3 infecting multidrug-resistant *Enterobacter aerogenes*. Front. Microbiol..

[21] Kim J (2021). Isolation, characterization, and genomic analysis of the novel T4-like bacteriophage PhiCJ20. Food Sci. Biotechnol..

[22] Morgan G, Lim D, Wong P, Tamboline B (2019). Electron Microscopy to visualize T4 bacteriophage interactions with *Escherichia coli* stain DFB1655 L9, an isogenic derivative of stain MG1655 engineered to express O16 antigen. UJEMI.

[23] Zhu Z, Tain R, Rhodes C (2003). A study of the decomposition behaviour of 12-tungstophosphate heteropolyacid in solution. Can. J. Chem..

[24] Smith BJ, Patrick VA (2004). Quantitative determination of aqueous dodecatungstophosphoric acid speciation by NMR spectroscopy. Aust. J. Chem..

[25] Takahashi K, Sano T, Sadakane M (2014). Preparation and characterization of Preyssler-type phosphotungstic acid, H_15__−__n_[P_5_W_30_O_110_M^n+^], with different encapsulated cations (M = Na, Ca, Bi, Eu, Y, or Ce), and their thermal stability and acid catalyst properties. Z. Anorg. Allgem. Chem..

[26] Hayashi A (2015). Cation effect on formation of preyssler-type 30-tungsto-5-phosphate: Enhanced yield of Na-encapsulated derivative and direct synthesis of Ca- and Bi-encapsulated derivatives. Z. Anorg. Allgem. Chem..

[27] Shitamatsu K (2021). Structural characterization of cerium-encapsulated Preyssler-type phosphotungstate: Additional evidence of Ce(III) in the cavity. Z. Anorg. Allgem. Chem..

[28] Casjens SR, Hendrix RW (2015). Bacteriophage lambda: Early pioneer and still relevant. Virology.

[29] Cuervo A (2014). Direct measurement of the dielectric polarization properties of DNA. Proc. Natl. Acad. Sci. USA..

[30] Harris JR (1999). Application of the negative staining technique to both aqueous and organic solvent solutions of polymer particles. Micron.

[31] Carlson K, Kutter E, Sulakvelidze A (2005). Appendix—Working with Bacteriophages: Common Techniques and Methodological Approaches. Bacteriophages: Biology and Applications.

[32] Kawato Y, Nakai T (2012). Infiltration of bacteriophages from intestinal tract to circulatory system in goldfish. Fish Pathol..

