# Supplementary material for: Preyssler-type phosphotungstate is a new family of negative-staining reagents for the TEM observation of viruses
Source: Sci Rep. 2022 May 12;12:7554. doi: 10.1038/s41598-022-11405-3 (PMC9098496; doi:10.1038/s41598-022-11405-3)
Supplement: Supplementary file 1 — Supplementary Figures. [file 41598_2022_11405_MOESM1_ESM.doc]

**Supplementary Information**

**Preyssler-type Phosphotungstate is a New Family of Negative-Staining Reagents for the TEM Observation of Viruses**

Koichi Sahiro^1^, Yasuhiko Kawato^2^, Kanae Koike^3^, Tsuneji Sano^1^, Toshihiro Nakai^4^, and Masahiro Sadakane^1*^

^1^Department of Applied Chemistry, Graduate School of Advanced Science and Engineering, Hiroshima University, 1-4-1 Kagamiyama, Higashi-Hiroshima, 739-8527, Japan

^2^Pathology Division, Nansei Field Station, Fisheries Technology Institute, Japan Fisheries Research and Education Agency, Minami-Ise, Mie 516-0193, Japan

^3^Natural Science Center for Basic Research and Development, Hiroshima University, 1-4-2 Kagamiyama, Higashi-Hiroshima, 739-8527, Japan

^4^Takehara Marine Science Station, Graduate School of Integrated Science for Life, Hiroshima University, Takehara, 725-0024, Japan


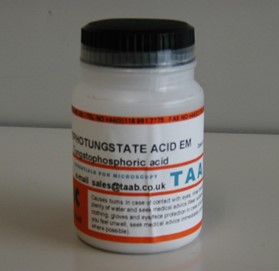


**Figure S1. (Top)** Commercial phosphotungstic acid (PTA) purchased from TAAB Laboratories Equipment Ltd. **(Bottom)** Infrared (IR) spectrum of commercial PTA. IR-band positions indicate that the PTA is the Keggin type.

**Figure S2.** Phosphorus-31 nuclear magnetic resonance (^31^P NMR) spectra of commercial phosphotungstic acid (PTA, purchased from TAAB Laboratories Equipment Ltd) in an aqueous solution with varying pH. **(a)** PTA was dissolved in water (0.33 M, pH 0.6). The ^31^P NMR chemical shift in water without NaOH addition indicates that the PTA was the Keggin-type. **(b)–(f)** Aqueous 1 M NaOH solution was added to the PTA solution (0.33 M, pH was 0.6) to change the pH to **(b)** 3.0, **(c)** 5.0, **(d)** 6.0, **(e)** 7.0, and **(f)** 7.5.

^31^P NMR spectra were recorded on a Varian system 500 (500 MHz) spectrometer (Agilent) (P resonance frequency: 202.333 MHz) where the spectra were referenced to an external 85% H_3_PO_4_ (0 ppm).


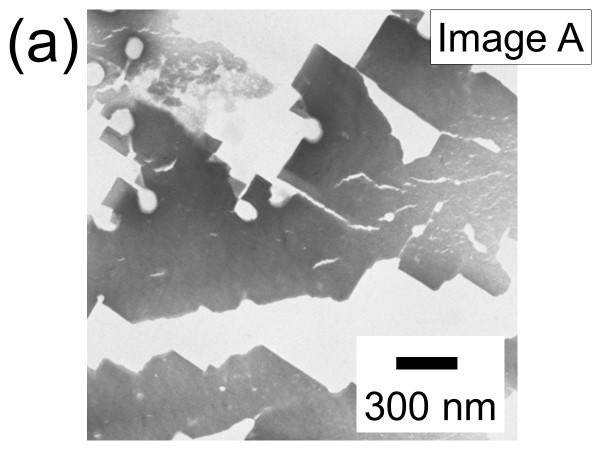

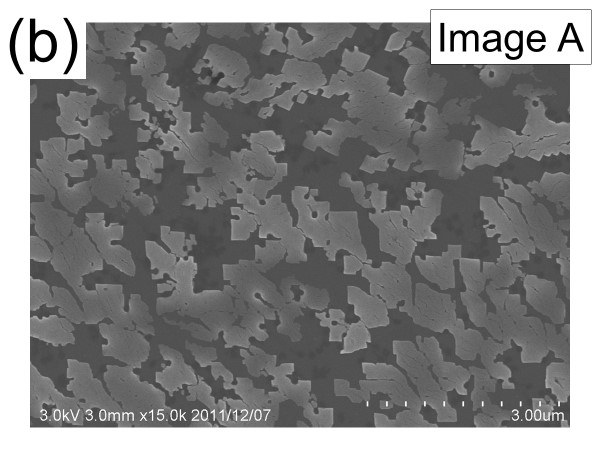

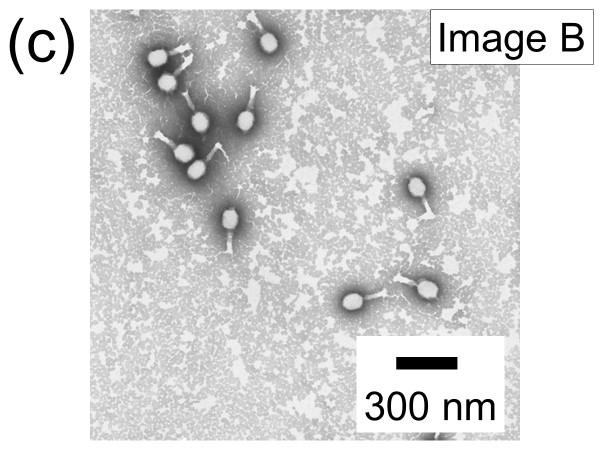

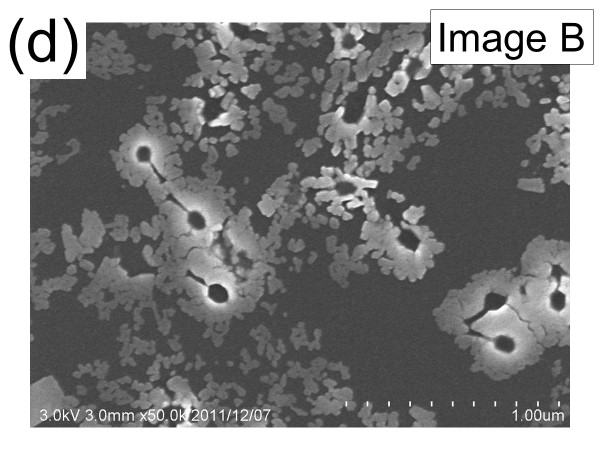

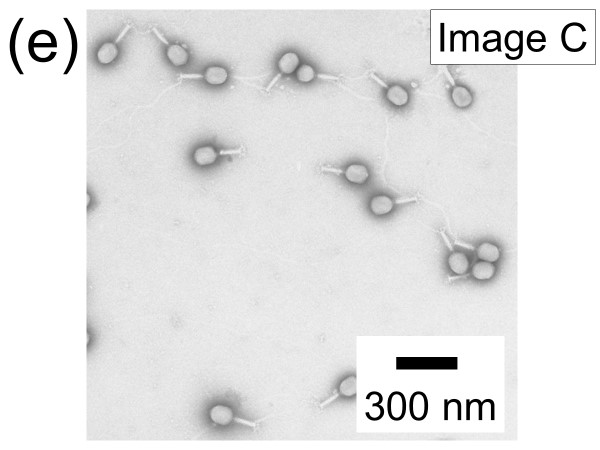

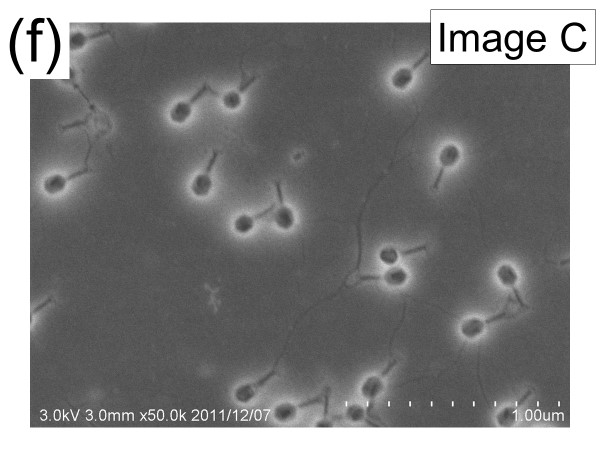

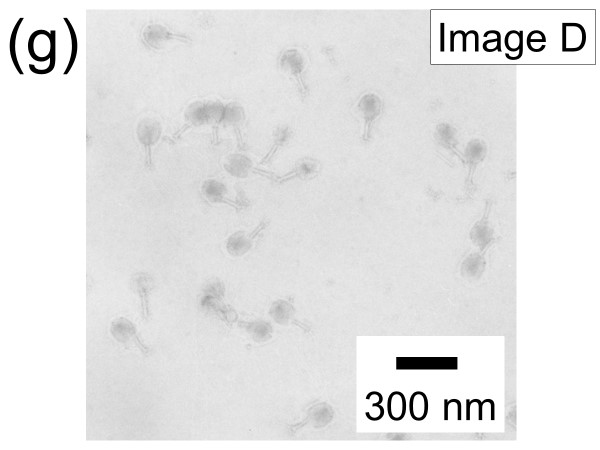

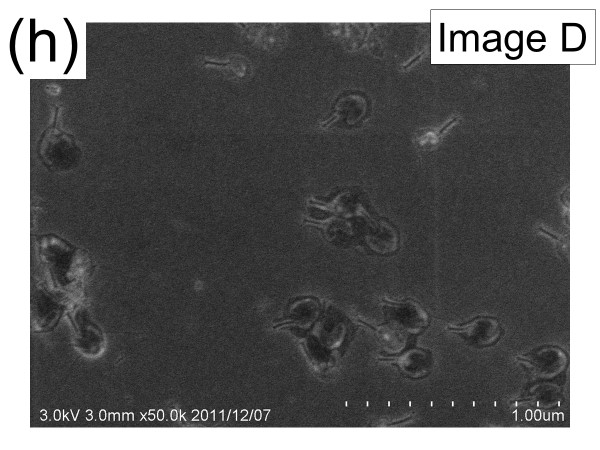


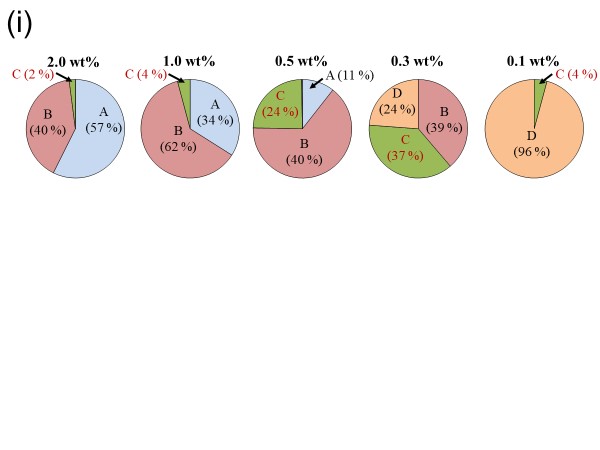


**Figure S3.** Effect of the concentration of Preyssler-type K_14_[P_5_W_30_O_110_Na(H_2_O)] as a negative staining reagent. Transmission electron microscopy (TEM) images (**(a), (c), (e),** and **(g)**) and scanning electron microscopy (SEM) images (**(b), (d), (f),** and **(h)**) of T4 phages with different concentrations of Preyssler-type K_14_[P_5_W_30_O_110_Na(H_2_O)]. **Image A**: Growth of a large crystal of K_14_[P_5_W_30_O_110_Na(H_2_O)]; the crystalline solid covered T4 phages, and shapes of the phages could not be observed in detail. **Image B**: K_14_[P_5_W_30_O_110_Na(H_2_O)] coated the T4 phages and parts of the carbon film; the long tail fibers were not observed. **Image C**: K_14_[P_5_W_30_O_110_Na(H_2_O)] coated the T4 phages; the long tail fibers were observable. **Image D**: The amount of K_14_[P_5_W_30_O_110_Na(H_2_O)] around the T4 phages was low and shape of phages was unclear.

**(i)** Pie-charts indicate the area of images A–D with different contents (wt.%) of Preyssler-type K_14_[P_5_W_30_O_110_Na(H_2_O)]. The copper grid contained 200 holes where phages were observed; we observed all holes and evaluated images A–D from each hole.


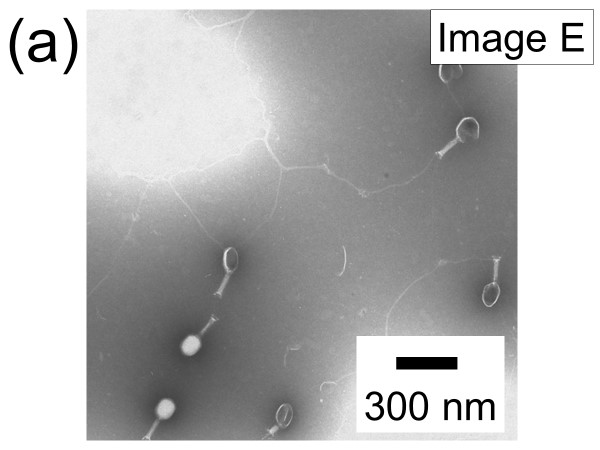

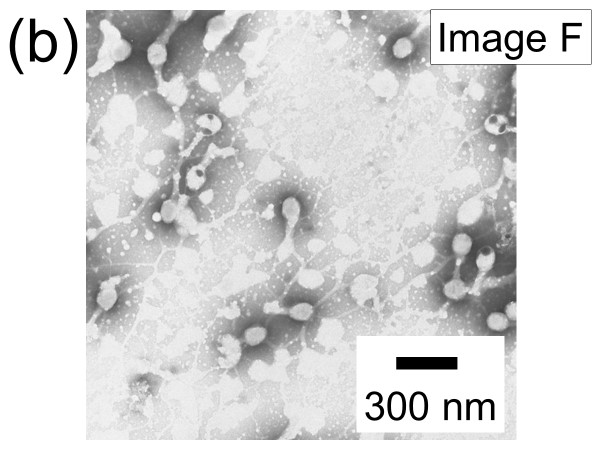

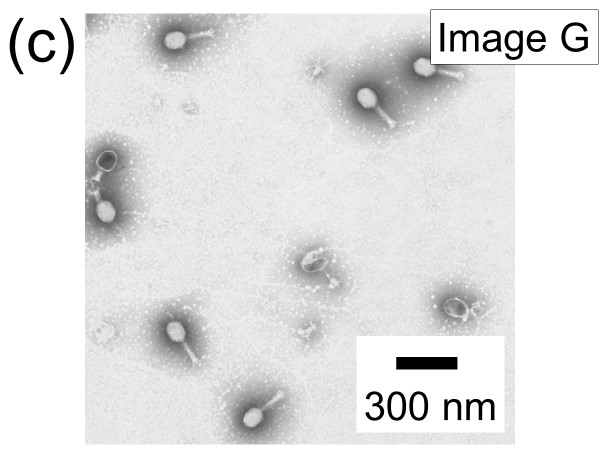

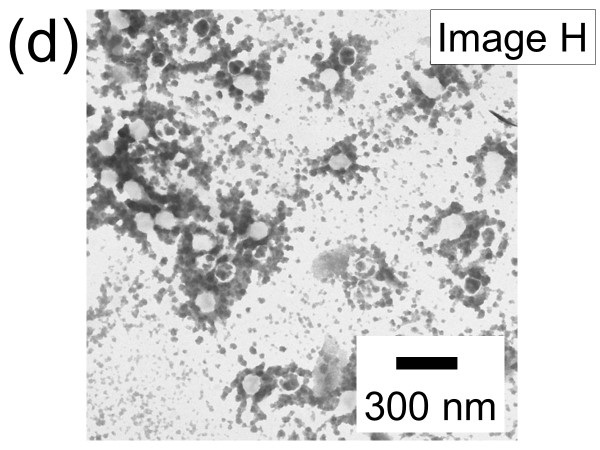


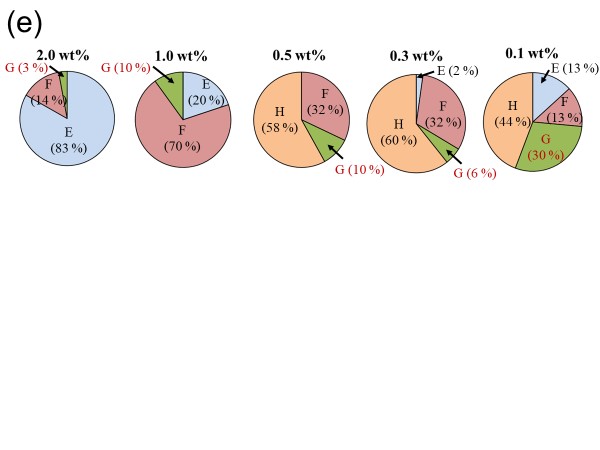


**Figure S4.** Effect of concentration of Keggin-type H_3_[PW_12_O_40_], after neutralization with KOH, as a negative staining reagent. Transmission electron microscopy (TEM) images (**(a), (b), (c),** and **(d)**) of T4 phage with different concentrations of Keggin-type H_3_[PW_12_O_40_] after neutralization with KOH. **Image E**: Solid covered T4 phages and large parts of the carbon film; long tail fibers were not observed. **Image F**: Solid coated T4 phages and parts of the carbon film; shapes of the phages were not observable in detail. **Image G**: Staining reagent coated T4 phages; long tail fibers were unclear. **Image H**: Staining reagents formed particles around T4 phages; shapes of the phages were unclear. **(e)** Pie-charts indicate the area of images E–H with different contents (wt.%) of Keggin-type H_3_[PW_12_O_40_]. The copper grid contained 200 holes where phages were observed; we examined all holes and evaluated images E–H from each hole.

Figure S5. Atomic force microscopy (AFM) image of T4 phage from image area C on the TEM grid on transmission electron microscopy (TEM) grid after TEM observation using Preyssler-type phosphotungstate and K_14_[P_5_W_30_O_110_Na(H_2_O)] as staining reagents. Height and length analyses are also shown.

Figure S6. pH vs. base/proton concentration ratio, [KOH]/[H^+^]. (●) H_14_[P_5_W_30_O_110_Na(H_2_O)] or (□) H_3_[PW_12_O_40_] was dissolved in water with [H^+^] = ~0.1 M, and 1.0 M NaOH was added to the solution.

**Figure S7.** Transmission electron microscopy (TEM) images of T4 phages using Preyssler-type phosphotungstates with different counter-cations: **(a)** and **(b)** Li-salt (Li_14_[P_5_W_30_O_110_Na(H_2_O)]); **(c)** and **(d)** Na-salt (Na_14_[P_5_W_30_O_110_Na(H_2_O)]); **(e)** and **(f)** NH_4_-salt ((NH_4_)_14_[P_5_W_30_O_110_Na(H_2_O)]); **(g)** tetrabutylammonium-salt ((Bu_4_N)_14_[P_5_W_30_O_110_Na(H_2_O)]); **(h)** tetrabutylphosphonium-salt ((Bu_4_P)_14_[P_5_W_30_O_110_Na(H_2_O)]).

All salts were prepared by adding base (AOH) solutions to H_14_[P_5_W_30_O_110_Na(H_2_O)] (0.84 g, 0.1 mmol) dissolved in 10 mL of water until the pH of the solution was ~6–7 (Table S1). The solution was then evaporated using a rotary evaporator. The relevant reaction is:

H_14_[P_5_W_30_O_110_Na(H_2_O)] + 14AOH = A_14_[P_5_W_30_O_110_Na(H_2_O)] + 14 H_2_O

**Table S1.** AOH, pH, and appearance of solution after the addition of AOH

| Salt | AOH solution | pH after addition of AOH | Appearance of solution | | Amount obtained [g] |
| --- | --- | --- | --- | --- | --- |
|  |  |  | After one drop of AOH | After addition of AOH |  |
| Li-salt | 2 M LiOH | 6.25 | Colorless, transparent | Colorless, transparent | 0.80 |
| Na-salt | 2 M NaOH | 7.27 | Colorless, transparent | Colorless, transparent | 0.74 |
| NH_4_-salt | 1 M (NH_4_)_2_CO_3_ | 6.07 | Colorless, transparent | Colorless, transparent | 0.75 |
| Bu_4_N-salt | 10 wt.% Bu_4_NOH | 6.97 | Turbid | Colorless, transparent | 0.92 |
| Bu_4_P-salt | 40 wt.% Bu_4_POH | 6.99 | Turbid | Slightly turbid | 0.93 |

**Figure S8.** Transmission electron microscopy (TEM) images of T4 phages using, as negative staining reagents, Preyssler-type phosphotungstate with various encapsulated cations: **(a)** Na^+^ (K_14_[P_5_W_30_O_110_Na(H_2_O)]), **(b)** Ca^2+^ (K_13_[P_5_W_30_O_110_Ca(H_2_O)]), **(c)** Y^3+^ (K_12_[P_5_W_30_O_110_Y(H_2_O)]), **(d)** Bi^3+^ (K_12_[P_5_W_30_O_110_Bi(H_2_O)]), **(e)** Ce^3+^ (K_12_[P_5_W_30_O_110_Ce(H_2_O)]), and **(f)** Eu^3+^ (K_12_[P_5_W_30_O_110_Eu(H_2_O)]). Concentration of staining reagent was 0.3 wt.% in water.

**(g)** Pie-chart indicating area of images A–D using 0.3 wt.% of K_12_[P_5_W_30_O_110_Eu(H_2_O)] as a negative staining reagent. The copper grid contained 200 holes where phages could be observed; we examined all holes and evaluated images A–D from each hole. **Image A**: Growth of a large crystal; crystalline solid covered T4 phages; shapes of phages not observable. **Image B**: K_12_[P_5_W_30_O_110_Eu(H_2_O)] coated T4 phages and parts of the carbon film; long tail fibers were not observable. **Image C**: K_12_[P_5_W_30_O_110_Eu(H_2_O)] coated T4 phages; long tail fibers were observed. **Image D**: Amount of K_12_[P_5_W_30_O_110_Eu(H_2_O)] around T4 phages was low; shapes of phages were unclear.
